# Fibrodysplasia Ossificans Progressiva: What Have We Achieved and Where Are We Now? Follow-up to the 2015 Lorentz Workshop

**DOI:** 10.3389/fendo.2021.732728

**Published:** 2021-11-10

**Authors:** Ruben D. de Ruiter, Bernard J. Smilde, Gerard Pals, Nathalie Bravenboer, Petra Knaus, Ton Schoenmaker, Esmée Botman, Gonzalo Sánchez-Duffhues, Maurizio Pacifici, Robert J. Pignolo, Eileen M. Shore, Marjolein van Egmond, Hans Van Oosterwyck, Frederick S. Kaplan, Edward C. Hsiao, Paul B. Yu, Renata Bocciardi, Carmen Laura De Cunto, Patricia Longo Ribeiro Delai, Teun J. de Vries, Susanne Hilderbrandt, Richard T. Jaspers, Richard Keen, Peter Koolwijk, Rolf Morhart, Jan C. Netelenbos, Thomas Rustemeyer, Christiaan Scott, Clemens Stockklausner, Peter ten Dijke, James Triffit, Francesc Ventura, Roberto Ravazzolo, Dimitra Micha, Elisabeth M. W. Eekhoff

**Affiliations:** ^1^ Department of Internal Medicine, Section Endocrinology, Amsterdam University Medical Center (Amsterdam UMC), Vrije Universiteit Amsterdam, Amsterdam Movement Sciences, Amsterdam, Netherlands; ^2^ Department of Clinical Genetics and Bone Histomorphology, Amsterdam University Medical Center (Amsterdam UMC), Vrije Universiteit Amsterdam, Amsterdam, Netherlands; ^3^ Department of Clinical Chemistry, Amsterdam University Medical Center (Amsterdam UMC), Vrije Universiteit Amsterdam, Amsterdam Movement Sciences, Amsterdam, Netherlands; ^4^ Freie Universität Berlin, Institute for Chemistry and Biochemistry, Berlin, Germany; ^5^ Department of Periodontology, Academic Centre for Dentistry Amsterdam (ACTA), University of Amsterdam and Vrije Universiteit, Amsterdam, Netherlands; ^6^ Department of Cell and Chemical Biology, Leiden University Medical Center, Leiden, Netherlands; ^7^ Translational Research Program in Pediatric Orthopaedics, Abramson Research Center, Division of Orthopaedic Surgery, The Children’s Hospital of Philadelphia, Philadelphia, PA, United States; ^8^ Department of Medicine, Mayo Clinic, Rochester, MN, United States; ^9^ Department of Orthopaedic Surgery and Genetics, and the Center for Research in FOP and Related Disorders, Perelman School of Medicine at the University of Pennsylvania, Philadelphia, PA, United States; ^10^ Department of Molecular Cell Biology and Immunology, Cancer Center Amsterdam, Amsterdam University Medical Center (Amsterdam UMC), Vrije Universiteit Amsterdam, Amsterdam, Netherlands; ^11^ Division of Biomechanics, Department of Mechanical Engineering, Katholieke Universiteit (KU) Leuven, Leuven, Belgium; ^12^ Prometheus division of skeletal tissue engineering, Katholieke Universiteit (KU) Leuven, Leuven, Belgium; ^13^ Department of Orthopaedic Surgery and Medicine, Center for Research in FOP and Related Disorders, The Perelman School of Medicine at the University of Pennsylvania, Philadelphia, PA, United States; ^14^ Department of Endocrinology and Metabolism, and the Institute for Human Genetics, Department of Medicine, University of California, San Francisco, CA, United States; ^15^ Division of Cardiovascular Medicine, Department of Medicine, Brigham and Women’s Hospital, Harvard Medical School, Boston, MA, United States; ^16^ Department of Neurosciences, Rehabilitation, Ophthalmology, Genetics, Maternal and Child Health (DINOGMI), Università degli Studi di Genova, Medical Genetics Unit, IRCCS Istituto Giannina Gaslini, Genova, Italy; ^17^ Rheumatology Section, Department of Pediatrics, Hospital Italiano de Buenos Aires, Buenos Aires, Argentina; ^18^ Teaching and Research Institute of the Hospital Israelita Albert Einstein, Sao Paulo, Brazil; ^19^ Berlin-Brandenburg Center for Regenerative Therapies, Charité Medical University of Berlin, Berlin, Germany; ^20^ Laboratory for Myology, Faculty of Behavioural and Movement Sciences, Vrije Universiteit Amsterdam, Amsterdam Movement Sciences, Amsterdam, Netherlands; ^21^ Centre for Metabolic Bone Disease, Royal National Orthopaedic Hospital, Stanmore, United Kingdom; ^22^ Department of Physiology, Amsterdam University Medical Center (Amsterdam UMC), Vrije Universiteit Amsterdam, Amsterdam, Netherlands; ^23^ Department of Pediatrics, Garmisch-Partenkichen Medical Center, Garmisch-Partenkirchen, Germany; ^24^ Department of Dermatology, Amsterdam University Medical Center (AmsterdamUMC), Vrije Universiteit Amsterdam, Amsterdam, Netherlands; ^25^ Division of Paediatric Rheumatology, Departmet of Paediatrics and Child Heath, Red Cross War Memorial Children’s Hospital, University of Cape Town, Cape Town, South Africa; ^26^ Botnar Research Centre, University of Oxford, Oxford, United Kingdom; ^27^ Departamento de Cièncias Fisiológicas, Facultad de Medicina y Ciencias de la Salud, Universitat de Barcelona, Barcelona, Spain

**Keywords:** fibrodysplasia ossificans progessiva (FOP), trials, therapy, disease models, inflammation, angiogenesis

## Abstract

Fibrodysplasia ossificans progressiva (FOP) is an ultra-rare progressive genetic disease effecting one in a million individuals. During their life, patients with FOP progressively develop bone in the soft tissues resulting in increasing immobility and early death. A mutation in the *ACVR1* gene was identified as the causative mutation of FOP in 2006. After this, the pathophysiology of FOP has been further elucidated through the efforts of research groups worldwide. In 2015, a workshop was held to gather these groups and discuss the new challenges in FOP research. Here we present an overview and update on these topics.

## Introduction

Fibrodysplasia ossificans progressiva (FOP) is an ultra-rare progressive genetic disease characterized by heterotopic ossification (HO) of muscles, tendons and ligaments, often preceded by periodic painful soft tissue swellings called flare-ups. During their lives, patients develop a “second” skeleton, resulting in increasing immobility and early death often due to thoracic insufficiency, infectious diseases, and traumatic falls (1).

**Figure 1:**
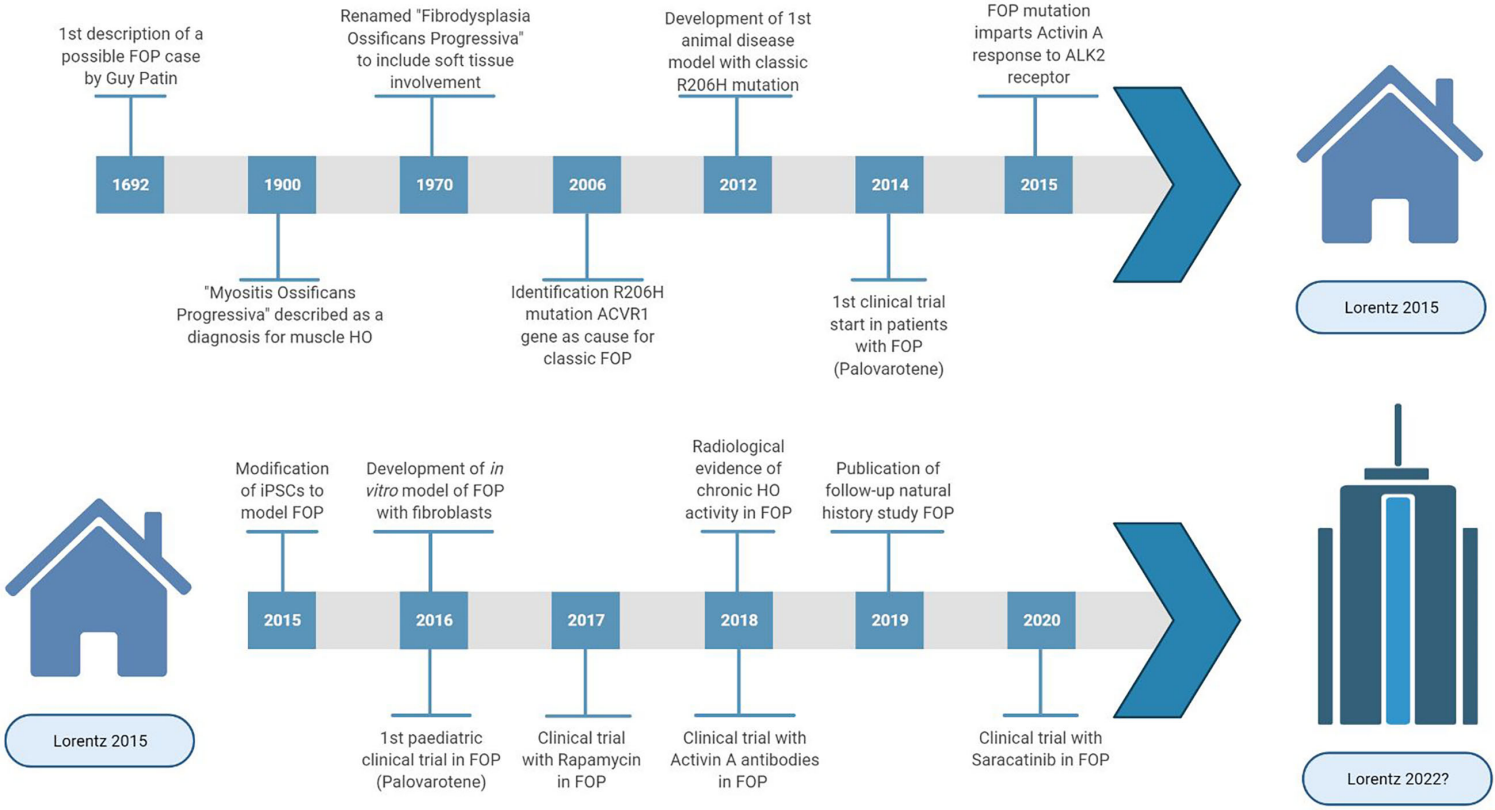
Highlights and key discoveries in FOP research leading up to the 2015 Lorentz meeting and after.

Progress of FOP research (**Figure 1**) has been slow due to three main factors. Firstly, obtaining tissue samples to examine the pathophysiology is difficult. Biopsies are contraindicated because of the increased risk for flare-ups in FOP. Secondly, FOP is frequently misdiagnosed, and so systematic data on early pathophysiology has been difficult to obtain. Finally, for a long time there were no cell or animal models for FOP as the causative genetic mutation was unknown. In 2006, the genetic cause of FOP was identified to be a missense mutation (R206H) in the *ACVR1* gene encoding the activin receptor-like kinase (ALK2) (2). The mutation induces hyperactivity of the ALK2 in response to bone morphogenetic protein (BMP) ligands as well as constitutive activity in the absence of ligands (3, 4). Also, while activing A induces ALK4-mediated canonical SMAD 2/3 signaling, the mutated ALK2 causes activin A to induce SMAD 1/5/9 signaling too, resulting in a skeletogenic signal instead of the usual response to activin A (5).

To date, there are no approved treatments to stop or reverse this disease, no biomarker to quantify FOP activity and many unanswered questions regarding pathophysiology.

In 2015, a Lorentz workshop was held, bringing international experts with a range of scientific backgrounds relevant to FOP research together for a week of scientific workshops discussing complex research problems and stimulating new initiatives for FOP treatment. Here we provide a comprehensive survey about the recent developments of basic and translational research on FOP.

## Identification of Heterotopic Ossification Progenitor Cells

HO is a complex, multi-stage process involving various cell types (6), but the exact progenitor cells that form the heterotopic bone are yet to be identified. Multiple populations of progenitor cells associated with muscle tissue have demonstrated osteogenic differentiation. Muscle stem cells (MuSCs) are muscle-resident stem cells essential for muscle growth and regeneration (7) and were initially a leading candidate for the HO progenitor cell. However, *in vivo* lineage tracing studies have shown that these cells do not significantly contribute to BMP-induced HO (8, 9), strongly arguing against MuSCs inducing HO in FOP (10).

Endothelial cells (EC) have also been proposed as a progenitor cell candidate. The endothelial marker Tie2 has been found in chondrocytes and osteoblasts in histological examination of HO tissues from individuals with FOP (11) and lineage tracing studies have identified Tie2 expression in roughly half the chondrocytes and osteocytes in heterotopic bone (11). However, Tie2 is not specific to ECs and more than 90% of the Tie2+ cells found in heterotopic bone are also platelet-derived growth factor receptor (PDGFR)α^+^Sca1^+^ indicating a mesenchymal rather than an endothelial origin (12).

In fact, these markers are also present in fibro/adipogenic progenitors (FAPs), another muscle tissue-resident progenitor. Cre/lox lineage tracing showed that FAPs can cause injury-induced and spontaneous HO in a FOP mouse model, greatly dependent on activin A signaling (13). Given the complexity of bone formation, perhaps cells from multiple origins are present and involved in ultimately forming the heterotopic bone.

## Inflammatory Triggers of HO

The contribution of the immune system in FOP is a keen focus of research. HO lesions harbor many cells of the immune system, such as lymphocytes, macrophages and mast cells (14, 15). Depletion of mast cells and macrophages have been reported to reduce HO volume in a FOP mouse model (16). The role of macrophages in HO has been investigated in different *in vivo* models with differing results (17, 18), leading to the idea that macrophage populations in FOP lesions are more heterogeneous than often presumed and may be responding to different types of injury signals.

The ALK2 mutation is also present in other cell types. Thus, the mutated ALK2 likely also affects immune responses. ECSIT (Evolutionarily Conserved Signal Intermediate in the Toll pathway) has been reported as a possible mechanism linking toll-like receptor activation in the innate immune response to aberrant SMAD signaling in FOP (19).

Blood samples taken from patients with FOP without symptoms of a flare-up have shown significantly elevated levels of pro-inflammatory interleukins indicating that patients with FOP may be in a constant pro-inflammatory state. Monocytes derived from patients with FOP, when stimulated with lipopolysaccharide, showed prolonged and increased cytokine and chemokine secretion, and prolonged activation of nuclear factor (NF)-κB (20, 21). A study of peripheral blood mononuclear cells from patients with FOP showed increased expression levels of DNAX accessory molecule-1 (DNAM-1) in monocytes, suggesting a functional effect in monocyte migration, and could represent a biomarker for the inflammatory state in FOP (22). Monocytes are also precursors for circulating osteogenic cells found in FOP lesions (23).

The hypoxic condition in inflamed tissues is another factor contributing to FOP pathogenesis, possibly through hypoxia inducible factor-1-α (HIF-1-α) which has been reported to promote amplification of BMP signaling through retention of the mutated ALK2 receptor in signaling endosomes (24). The fibroproliferative stage with extracellular matrix production that normally occurs after injury also appears to be overactive in FOP, leading to tissue stiffening and increased mechano-sensitivity in favor of osteogenic processes (25).

## Vascularization in FOP

Angiogenesis is an important process involved in the development of FOP lesions. The inflammation, soft tissue destruction, and subsequent infiltration of immune cells all depend on vascularization. In the fibroproliferative phase the inflamed tissue is infiltrated by chondrocytes promoting a proteoglycan-enriched environment, which becomes progressively hypoxic. Hypoxic conditions favor chondrocyte differentiation partially by sustaining BMP signaling activation (24), and induce expression of vascular endothelial growth factor (VEGF), promoting the infiltration of blood vessels, which in turn drives endochondral bone formation. Interestingly, monocytes isolated from FOP patients showed increased VEGF secretion upon an inflammatory trigger compared to controls (18).

BMP and VEGF signaling play key roles in regulating blood vessel homeostasis; gene mutations in components of the BMP signaling pathway are associated with cardiovascular conditions (26), and disturbances in the angiogenesis-osteogenesis axis can cause bone disorders (27). Whether the mutant FOP ALK2 also disturbs EC function through aberrant BMP signaling is currently under investigation.

Angiogenesis is initiated by the formation of tip cells supported by proliferating stalk cells to forming new sprouts from pre-existing vessels. This process is coordinated by VEGF-, BMP2- and BMP6 signaling. During angiogenesis, BMP2 primarily signals through ALK3, whereas BMP6 signals through ALK2. Upon ALK2 knockdown, hypersprouting was observed in *in vitro* EC models, whereas ALK3 knockdown appeared to have the opposite effect (28). Recent data showed that EC’s derived from human induced pluripotent stem cells (hiPSC) follow the same principle and hiPSCs derived from patients with FOP show activin A induced SMAD 1/5 signaling (29).

Vascular leakage and edema have also been described in HO lesions in FOP (30). BMP6 stimulation in ECs causes internalization of VE-cadherin changing the endothelial architecture. VE-cadherin in turn appears to interact with ALK2 in a ligand-dependent manner by stabilizing the BMP receptors in the EC junctions (31). ECs from patients with FOP appear to have decreased expression of vascular endothelial (VE)-cadherin under inflammatory conditions (32), possibly due to an altered interaction of VE-cadherin signaling with the mutated ALK2 receptor complex.

## Suitability of FOP Disease Models

Since the discovery of the mutation (2), several cellular and animal models have been developed to examine the effects of FOP ALK2 mutations on BMP signaling and chondro/osteogenesis.

Availability of human cell models is limited due to restrictions on obtaining patient material and our incomplete knowledge of the progenitor cell types relevant to FOP HO. Dermal fibroblasts derived from patients with FOP have been successfully transdifferentiated to cells of an osteogenic lineage (33). Periodontal ligament fibroblasts have also been isolated and induced to osteogenesis and osteoclastogenesis (34). hiPSCs obtained from patients with FOP are able to differentiate to ECs (29, 35, 36) and pericytes with increased mineralization, but did not develop into mature osteoblasts (36). Connective tissue progenitor cells from discarded primary teeth have been used to examine the effects of FOP mutations on BMP signaling and chondrogenic/osteogenic differentiation (19, 24, 37, 38). C2C12 myoblasts have been altered to express ALK2^R206H^ with doxycycline dependent promoter to simulate FOP (39).

A fruit fly model carrying the classical R206H mutation demonstrated over activation of BMP signaling by the ALK2^R206H^ receptor but also ligand independent signaling of the receptor (40), consistent with earlier *in vitro* analyses (41–43). An embryonic chicken model was used to study the role of several ALK2 mutations and demonstrated that the FOP ALK2^Q207E^ and ALK2^R206H^ mutation, along with the engineered constitutively active ALK2^Q207D^ mutation, caused FOP-like phenotypes with skeletal malformations and HO (44).

In mice, activating mutations in ALK2 are lethal during embryonic development (45), therefore investigations of the *in vivo* effects of ALK2 activating mutations have required either chimeric/mosaic expression of mutant cells or a conditional gene expression model. The first such mouse model, using a Cre-Lox inducible ALK^Q207D^ transgene was developed prior to the identification of ALK2 as relevant to FOP (45). Later, this model was used with adenovirus expressing Cre and tamoxifen-responsive Cre alleles to induce postnatal activation of the ALK2^Q207D^ transgene (46). Although the ALK2^Q207D^ substitution is not a naturally occurring FOP mutation in humans, these mouse models provided the first mammalian systems to study the effects of excessive BMP signaling by ALK2, importantly demonstrating a requirement for tissue injury and inflammation in addition to mutant ALK2 expression for the development of heterotopic bone (47).

Subsequently, researchers have developed mouse models harboring the common FOP ALK2^R206H^ mutation. A chimeric model with a variable proportion of cells expressing a heterozygous ALK2^R206H^ allele yielded intermittent live births, mimicking classic FOP features such as HO development in response to muscle injury, hind limb digit malformation, and joint fusions (48). A Cre-dependent knock-in model with inducible ALK2^R206H^ expression has been used to mimic HO formation in response to various injuries, highlighting the importance of activin A in ALK^R206H^ signaling function (49–52). The progression of HO formation in ALK2^R206H^ mouse models appears to closely reproduce the events of HO formation from an early-stage immune cell response to a robust fibroproliferative stage that transitions to endochondral ossification (15, 16, 48). These models also feature the distinct patterns of HO within the axial and extra-axial skeleton and exhibit both injury-dependent and spontaneous progression of HO (50, 52).

A zebrafish FOP model has also been developed and embryonic development assays have been used to investigate the mechanism through which mutant ALK2 receptors enhance BMP-phosphorylated (p)SMAD 1/5 signaling (53–55).

A novel approach is a computational disease model. Computational models of endochondral ossification have previously been developed (56, 57). In these models the interplay between growth factors, angiogenesis, oxygen, recruitment, proliferation and differentiation of osteoprogenitor cells can be considered. These models could be adapted to simulate endochondral ossification in FOP and provide an additional way to evaluate the effect of therapeutic interventions in FOP.

In summary, there are numerous *in vitro* and *in vivo* models available with the potential to further investigate and understand FOP. It will also be important to establish how closely these model systems reflect the pathophysiology of FOP in humans and how well they address the various complexities of the FOP phenotype. Acknowledging the advantages and disadvantages of each of these models can allow them to complement each other, maximizing the information gained in preclinical FOP research.

## Possible Targets for Therapy in FOP

**Figure 2:**
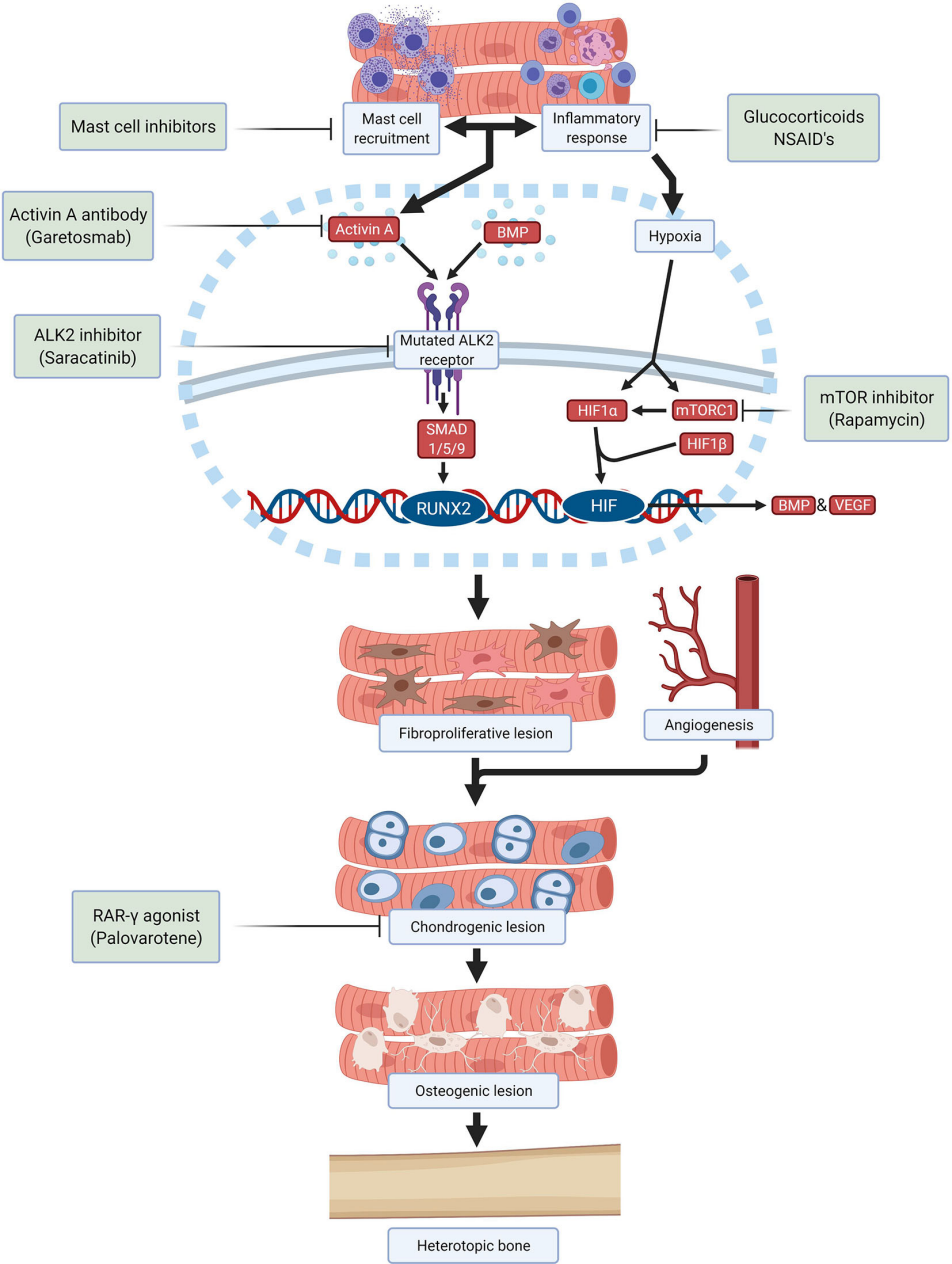
Schematic overview of drugs and investigational compounds currently used and/or evaluated in FOP treatment and their respective targets.

Despite many efforts, still there is no effective and specific treatment approved for FOP. Therapy is focused on treating flare-ups with glucocorticoids and nonsteroidal anti-inflammatory drugs upon presentation (58). Taking the different stages of HO in FOP into consideration it is possible to identify different processes which can be considered as targets to develop therapies by different approaches (**Figure 2**).

Saracatinib, a kinase inhibitor targeting src-family kinases originally developed as a treatment for various solid tumors, is a potent ALK2 inhibitor with efficacy against HO in preclinical models and is now being repositioned as a potential treatment for FOP in an ongoing phase 2 clinical trial (NCT04307953) (59). Several other ALK2 inhibitors have been developed with the goal of improving potency and selectivity for ALK2 receptor inhibition, with promising safety in phase 1 studies and are anticipated to advance to phase 2 efficacy studies in the near future.

Alternatively, the stimulation of the ALK2 receptor by ALK2 ligands can be prevented. A neutralizing antibody specific for activin A (garetosmab) has been evaluated in a phase 2 clinical trial (NCT03188666) after promising preclinical results (49). Recently, mTOR (mammalian target of rapamycin) has been identified as a key factor in the early hypoxic and inflammatory stages of HO (21). Besides its important immunoregulatory function, mTOR signaling is required for chondrogenesis and osteogenesis induction. Crosstalk between mTOR signaling and BMP signaling may amplify HO in FOP (60). In preclinical studies, rapamycin successfully inhibited HO in a mouse model and a clinical trial is being performed to evaluate its efficacy and safety in patients with FOP (UMIN000028429) (60, 61).

Downstream signaling initiated by activation of ALK2 also offers opportunities to prevent HO. Palovarotene, a retinoic acid receptor-gamma (RAR-γ) agonist, inhibits HO in FOP mouse models by blocking chondrogenic differentiation of the progenitor cells and is currently being investigated in multiple phase 2 and phase 3 trials (NCT02279095, NCT02190747, NCT03312634) (51, 62, 63). Other therapies being investigated are VEGF inhibitors, ligand traps, phosphoinositide 3-kinases (PI3K)-inhibitors, siRNAs, HIF1-α blockers and transforming growth factor-β activated kinase (TAK)1 inhibitors. Once a successful therapeutic strategy for preventing HO in FOP is available, surgical intervention may become feasible for excising heterotopic bone and restoring function.

## Clinical Trials in Ultra-Rare Diseases

Therapeutic development in FOP shares many challenges faced by other ultra-rare diseases such as a limited understanding of natural history to inform trial design, dearth of validated and surrogate outcome measures to quantify the disease during the limited time span of a clinical trial, and small numbers of patients available for clinical trials (64, 65).

The randomized controlled trial (RCT) is the gold standard for determining drug efficacy in a clinical trial setting. Randomization minimizes selection bias and distributes potential confounders between study groups. RCT power decreases rapidly with diminished smaller cohorts as inter-individual differences become more pronounced, increasing the risk of known and unknown covariates affecting the trial results.

An uncontrolled trial may be feasible when the natural history of a disease is well-established. In this design, the effect of the intervention can be compared against the natural history of the disease. In FOP however, the natural history of the disease is still being investigated and it is known that disease progression varies between individuals (66). Additionally, subject may report less adverse events in a non-interventional natural study than in an interventional clinical trial, creating bias against the drug. However, studies have been performed to mitigate this potential bias (67).

Both trial designs have their drawbacks but remain important options for determining drug efficacy in FOP. Future trials in FOP should acknowledge these disadvantages, implementing smart trial designs and statistical methods to address inherent limitations of a small and heterogeneous population, thus maximizing the information obtained while supporting patient safety (65, 68). There is an urgent need to establish an imaginative and equitable approach towards clinical trials in FOP given the multitude of drugs being developed and the limited number of patients.

## Determination of Disease Activity in FOP

Another problem that FOP faces is the difficulty to evaluate individual disease activity. Clinical symptoms of a flare-up such as pain, swelling, erythema and warmth are non-specific, and it is not possible to predict whether the acute phase will end up with HO or will resolve (66). A multitude of inflammatory, chondrogenic and osteogenic bone markers have been investigated, and although some were markedly elevated in patients with FOP, none have shown an association with disease activity or been able to predict HO formation adequately (69–71).

Conventional imaging techniques are only able to detect HO after formation of bone tissue. MRI (magnetic resonance imaging) and ultrasonography are suitable to detect soft tissue edema associated with the inflammatory stage of HO but are non-specific and unable to reveal bone formation (72, 73). Nucleotide imaging such as the [^18^F]-sodium fluoride (NaF) PET (positron emission tomography) scan can detect bone formation before it is visible on conventional CT (computed tomography). Interestingly, PET/CT and MRI scanning revealed that not every flare-up resulted in HO and showed continuous FOP activity not related to a flare-up (74).

Determination of disease activity with a suitable biomarker and imaging techniques is necessary for evaluation of potential therapies in FOP. A combination of markers may be needed to reflect the multiple stages of HO in FOP; ongoing efforts exist on FOP biomarker development (20).

## Discussion and Future Research

Looking back at the topics discussed in 2015, the meeting identified key issues in which progress has been made through collaborative approaches (**Figure 1**). However, it is also clear that FOP research and treatment still face many challenges. Big questions remain regarding the pathophysiology of FOP such as the identity of the HO progenitor cell and the effect of the ALK2 mutation on the immune response and angiogenesis. Also, with the advent of clinical trials for FOP, it has become clear that we still need to obtain as much information as possible in the preclinical phase including cell and molecular mechanisms. This requires further use and development of *in vitro* and *in vivo* disease models, and perhaps exploring options such as computational modelling. During clinical trials, the information gained must be maximized through means of careful trial design and proper evaluation of disease activity. To achieve this in FOP, international collaboration is paramount and has to be fostered. Maybe the time is ripe to make a point and gather the FOP research community in a new meeting to share and discuss the most recent research strategies again.

## Data Availability Statement

The original contributions presented in the study are included in the article/supplementary material. Further inquiries can be directed to the corresponding authors.

## Author Contributions

RdR wrote the manuscript and created the figures with input from all authors. EE, GP, NB, PKn, DM, and RR organised the original workshop in which topics were discussed. GP, NB, PK, TS, GSD, MP, RP, ES, ME, HO, PY, RR, DM, EE, RB, CC, TV, SH, RJ, RK, PKo, RM, CN, PD, JT, and FV all attended the 2015 workshop, provided valuable contributions to the discussions and comments on the manuscript. RdR, BS, EB, TR, ES, FK, CSc, CSt, PD, and EH all provided valuable updates to the discussion of the research done since the 2015 meeting. All authors contributed to the article and approved the submitted version.

## Conflict of Interest

The authors declare that the research was conducted in the absence of any commercial or financial relationships that could be construed as a potential conflict of interest.

## Publisher’s Note

All claims expressed in this article are solely those of the authors and do not necessarily represent those of their affiliated organizations, or those of the publisher, the editors and the reviewers. Any product that may be evaluated in this article, or claim that may be made by its manufacturer, is not guaranteed or endorsed by the publisher.
